# Effect of Particle Size and Fiber Reinforcement on Unconfined Compressive Behavior of EICP-Cemented Recycled Fine Aggregate

**DOI:** 10.3390/ma19071440

**Published:** 2026-04-03

**Authors:** Meixiang Gu, Zhouyong Liu, Wenyu Liu, Jie Yuan

**Affiliations:** School of Civil Engineering and Transportation, Guangzhou University, Guangzhou 510006, China; mxgu@gzhu.edu.cn (M.G.); 2112316049@e.gzhu.edu.cn (Z.L.); yuanj@gzhu.edu.cn (J.Y.)

**Keywords:** recycled fine aggregate, enzyme-induced carbonate precipitation, particle gradation, fiber reinforcement, calcium carbonate precipitation, unconfined compressive strength

## Abstract

Against the backdrop of dual-carbon goals and resource constraints, the high-value utilization of recycled fine aggregates (RFAs) remains limited, leading to inconsistent engineering performance and insufficient durability. Enzyme-induced carbonate precipitation (EICP) represents a promising low-carbon cementation method, yet its deposition uniformity and cementation efficiency are influenced by the pore structure of granular media and associated mass transfer pathways. This study employs a two-stage experimental design to investigate the synergistic effects of particle size distribution characteristics, represented primarily by *d*_50_, and fiber addition on EICP-cemented RFA. Phase I (fiber-free; *d*_50_ = 0.67–1.14 mm) results indicate that, across the tested gradation schemes, the CaCO_3_ content generally decreased from 9.49% to 7.72% as the representative *d*_50_ increased, while the dry density changed only slightly (1.637–1.617 g/cm^3^). However, the unconfined compressive strength (UCS) decreased from 1000 kPa to 541 kPa (45.9% reduction), indicating that strength is primarily governed by the connectivity of the cementation network rather than solely by the degree of densification. In Phase II, glass fiber (GF), polypropylene fiber (PPF), and jute fiber (JF) were incorporated into the ERFA4 gradation scheme selected for fiber modification. All three systems exhibited a unimodal optimum pattern: the peak CaCO_3_ contents reached 10.71% (GF 0.5%), 10.11% (PPF 0.7%), and 11.46% (JF 0.7%), corresponding to peak UCS values of 1917, 1874, and 2450 kPa, respectively. Microscopic analysis suggested that fiber bridging coupled with CaCO_3_ deposition may contribute to the formation of a “fiber-CaCO_3_-particle” stress-transfer network, which is consistent with the observed enhancements in load-bearing capacity, ductility, and post-peak stability.

## 1. Introduction

Against the backdrop of dual-carbon goals and resource constraints, the high-value utilization of construction and demolition waste has become a shared concern in civil engineering materials and geotechnical engineering. Recycled fine aggregates (RFAs), with their abundant sources and relatively controllable processing costs, are considered to have significant potential as a substitute for natural sand. However, characteristics such as residual mortar adhesion, high porosity, irregular and angular particle morphology, and high or fluctuating water absorption often result in performance variability, weak interfacial bonding, and insufficient durability [1,2,3]. These issues restrict the widespread application of RFA in low-grade civil engineering applications such as unbound pavement base, embankment fill, and soil stabilization [4]. Extensive research and reviews further indicate that the high porosity and microcracks in adhered mortar can weaken the interfacial transition zone (ITZ), increase water absorption, reduce density, and enhance material heterogeneity, which may adversely affect the compaction behavior and service performance of recycled aggregates when used as engineering fill media [3,5,6,7,8].

Compared to cementitious materials, biomineralization consolidation technology has garnered attention due to its potential low-carbon attributes and controllable precipitation cementation mechanisms [9,10,11,12]. Among these, enzyme-induced carbonate precipitation (EICP) utilizes urease to catalyze urea hydrolysis and induce CaCO_3_ precipitation, forming bridging bonds in particle contact zones to enhance the strength and stiffness of the consolidated body [11,12]. Existing results indicate that EICP shows promising applications in sand consolidation, the improvement of homogeneity and stability, and environmental impact assessment, as well as crack repair and erosion protection for cementitious materials [11,12,13,14,15,16]. Recent reviews and studies have further highlighted that EICP/MICP performance is strongly governed by treatment strategy, precipitation uniformity, and activity regulation, while current research is increasingly extending biomineralization approaches to sustainable geomaterials and construction materials [9,12].

However, the engineering application of EICP still faces critical bottlenecks such as uneven precipitation and blockage. The reaction process is sensitive to parameters such as ambient temperature, solution pH, urease activity, and injection methods. This sensitivity often leads to rapid precipitation near the inlet or in localized areas, causing pore throats to become blocked by CaCO_3_ and reducing permeability. Consequently, subsequent reaction fluids are inhibited from flowing in, resulting in insufficient deep cementation. Ultimately, this reduces cementation efficiency and limits strength enhancement [17,18,19,20]. Furthermore, different enzyme-source systems and solidification processes exhibit significant variations in precipitation rate, crystal morphology, and uniformity, resulting in noticeable discrepancies in solidification effectiveness and strength enhancement [21,22,23]. To improve precipitation efficiency and structural stability, recent studies have employed strategies such as multiphase/stepwise injection, enzyme activity control, and admixtures to regulate nucleation, growth, and precipitation pathways, thereby enhancing CaCO_3_ distribution uniformity and macroscopic response stability [18,19,20,21,22]. Concurrently, ionic environments and mineralogical characteristics (e.g., Mg^2+^-mediated Mg-calcite formation) substantially influence MICP/EICP morphology and consolidation efficacy [24].

Beyond reaction kinetics, EICP cementation is strongly governed by the pore structure and flow pathways within granular media. Particle size and grading alter pore size distribution, specific surface area, and solution migration pathways, thereby influencing reactant transport, precipitation location, and bridging probability [25,26,27]. The median particle size *d*_50_ in grading comprehensively characterizes the dominant particle size fraction and corresponding pore geometry, and is therefore commonly used to correlate structure with consolidation response. Previous studies in natural sand or calcareous sand systems reveal that bio-cementation strength may exhibit non-monotonic responses to representative particle size and grading or show system-dependent variations. “Finer does not necessarily mean stronger,” which is linked to pore connectivity, transport-reaction matching, and the proportion of effective precipitation bridging [25,26,27,28]. However, RFA exhibits heterogeneous characteristics as a material with a “porous adherent layer with complex morphology,” whose pore structure, water absorption behavior, and interfacial state significantly differ from those of natural sand. In such systems, systematic evidence and interpretable frameworks for understanding the influence of particle size distribution characteristics, represented primarily by *d*_50_, on CaCO_3_ deposition efficiency, dry density development, and strength evolution remain lacking.

Furthermore, bio-mineralized consolidation materials generally tend to become more brittle. Fiber composites are employed to enhance post-peak load-bearing capacity and ductility through crack bridging, friction pull-out, and network confinement, potentially forming synergistic “fiber skeleton-mineral cement” structures with CaCO_3_ precipitation [29,30,31,32,33]. Previous studies indicate that incorporating fibers like polypropylene into bio-cemented sands significantly enhances ductility, toughness, and residual strength. More recent studies have further shown that the reinforcing effect depends not only on fiber inclusion itself, but also on fiber type, content, and length, as well as on how the fibers interact with CaCO_3_ precipitation distribution and the pore structure of the bio-cemented matrix [29,34,35]. Microscopically, this relates to bond promotion and energy dissipation through fiber-precipitate interactions [36]. Coupling natural jute fibers with MICP forms stable connections through strong fiber-CaCO_3_ bonding, restricting fracture propagation to enhance consolidated strength and mitigate brittle failure. Simultaneously, their adsorption and immobilization of spores demonstrate potential for bio-sand self-healing [34]. However, the effectiveness of fibers highly depends on the quality of matrix cementation and pore size matching. When precipitation distribution is uneven, or the pore size is incompatible with the fiber scale, diminishing returns may occur despite increased fiber content [22,26,32,33]. Therefore, it is preferable to first optimize the particle size distribution (mainly *d*_50_) to obtain a matrix suitable for uniform mineralization and effective precipitated bridging. Subsequent optimization of fiber combinations can then yield scalable parameters and clarify the underlying mechanisms.

Despite the increasing number of studies on EICP-treated granular media, and although recent studies have explored biomineralization-based modification of recycled aggregates and recycled aggregate concrete, the roles of particle-size-related characteristics and matrix heterogeneity in RFA systems remain insufficiently understood [37,38]. In particular, there is still a lack of a systematic framework that identifies an RFA gradation favorable for uniform EICP cementation before optimizing fiber reinforcement on that basis. Accordingly, this study focuses on EICP-cemented recycled fine aggregate rather than natural sand, thereby addressing a granular system with adhered mortar, high porosity, and heterogeneous interfacial characteristics. It further proposes a two-stage strategy that combines gradation-based matrix screening with subsequent fiber reinforcement optimization. In addition, macroscopic performance is interpreted in conjunction with microstructural evolution to clarify the roles of CaCO_3_ precipitation morphology, fiber bridging, and pore filling in strength development. In this respect, the study is expected to provide a more systematic basis for understanding the coupled influence of matrix gradation and fiber reinforcement in EICP-treated RFA.

To address these issues, a two-stage experimental design was employed to systematically investigate the effects of particle size distribution characteristics, represented primarily by *d*_50_, together with fiber type and dosage, on the performance of EICP-cemented RFA. In Phase I, four gradation schemes (ERFA1-ERFA4) with different particle size distributions were established under fiber-free conditions, with *d*_50_ used as the primary grouping indicator. Using CaCO_3_ content, dry density, and unconfined compressive strength as evaluation indicators, this phase identified the gradation condition most favorable for effective bonding and strength transfer under the present test conditions. In Phase II, based on the findings of Phase I, glass fiber, polypropylene fiber, and jute fiber were introduced at multiple dosage levels to evaluate their synergistic effects on strength and structural stability under the selected gradation condition. Finally, combined with scanning electron microscopy characterization, the study elucidates the microstructural mechanisms linking CaCO_3_ precipitation morphology, fiber bridging, and pore filling. The results are expected to provide useful guidance for the parameter design of EICP-based RFA stabilization, while also contributing to the understanding of how gradation characteristics affect cementation behavior and the effectiveness of fiber reinforcement.

## 2. Materials and Methods

### 2.1. Materials

#### 2.1.1. Soybean Crude Urease and Cementation Solution

All EICP treatment methods in this study utilized crude urease extracted from soybeans. The soybean powder was sourced from Xuzhou, China. To prepare the crude soybean urease solution, an appropriate amount of soybean powder was first dried in a 40 °C oven for 8 h to remove excess moisture. An appropriate amount of the dried soybean powder was then mixed with deionized water and stirred for 30 min to form a soybean powder solution with a concentration of 100 g/L. The mixture was subsequently placed at 4 °C and allowed to stand for 12 h to ensure complete dissolution of the urease from the soybean powder into the water. Next, the soybean powder solution was transferred into centrifuge tubes and centrifuged for 10 min at 4 °C and 8000 r/min. Finally, the supernatant was filtered through a geotextile to obtain the soybean urease solution.

Urea and anhydrous calcium chloride (CaCl_2_) solid reagents were purchased from Tianjin Zhonglian Chemical Reagent Co., Ltd. (Tianjin, China) and used as raw materials for preparing the binder solution. The corresponding masses of urea and CaCl_2_ were weighed according to a 1:1 molar ratio, dissolved in deionized water, and the volume was adjusted to the required amount to prepare an equimolar urea-calcium chloride binder solution. The urea concentration was 1.35 mol/L, and the calcium chloride concentration was 1.35 mol/L.

#### 2.1.2. Characteristics of Recycled Fine Aggregate

The RFA used in this study was sourced from the Guangzhou Huangpu District Construction Waste Comprehensive Recycling Demonstration Base (Guangzhou, China). Before testing, the RFA was cleaned to remove impurities and dried at 108 °C until constant weight. Subsequently, four gradation schemes were prepared using standard sieving methods. These schemes were primarily distinguished by their median particle sizes (*d*_50_ = 0.67, 0.83, 1.00, and 1.14 mm), while the corresponding coefficients of uniformity (*Cu*) and curvature (*Cc*) also varied among groups. To minimize the impact of initial compaction variations on bonding effectiveness, the initial void ratio (*e*) of all graded specimens was controlled at 0.75 ± 0.01, ensuring comparability of test results across different grading conditions.

**Figure 1:**
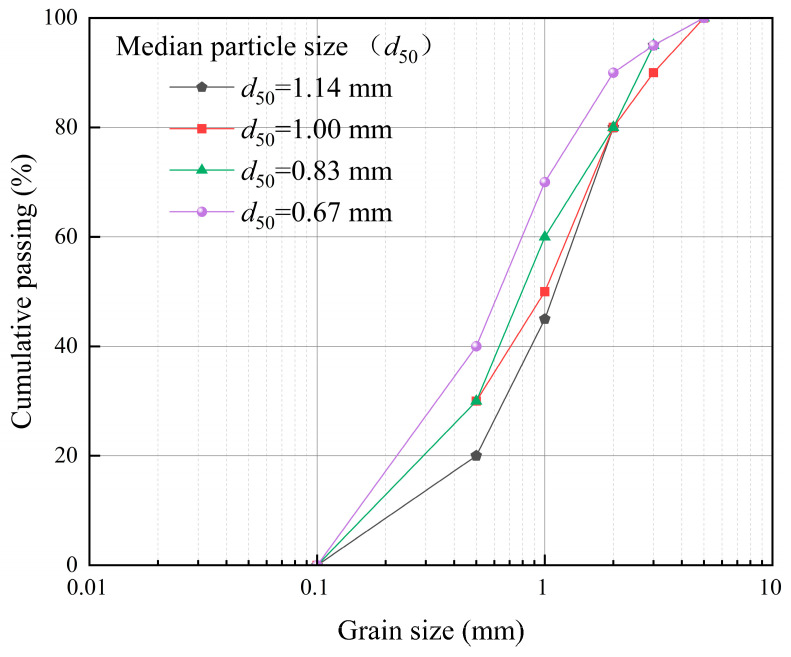
Grain-size distribution curves (cumulative passing) of recycled fine aggregates with different median particle sizes (*d*_50_).

To quantify the particle distribution characteristics of different gradation schemes, in addition to *d*_50_, both the coefficient of uniformity (*Cu*) and the coefficient of curvature (*Cc*) were employed to characterize the gradation (definitions are provided in the table notes). Furthermore, the basic physical properties of each RFA gradation—including specific gravity and maximum/minimum dry density—were determined as foundational parameters for subsequent specimen preparation and result analysis. Particle size distribution curves for different *d*_50_ gradations are shown in Figure 1. Relevant physical properties were determined according to GB/T 50123—2019 [39], with the results summarized in Table 1. This study employed *d*_50_ as the primary indicator for gradation grouping, while *Cu* and *Cc* were used to supplement the characterization of gradation distribution differences. Therefore, the observed responses should be interpreted as reflecting the effect of overall gradation characteristics rather than the independent effect of *d*_50_ alone. For clarity of presentation, the following discussion is organized mainly around the variation in *d*_50_.

#### 2.1.3. Fiber Types

**Figure 2:**
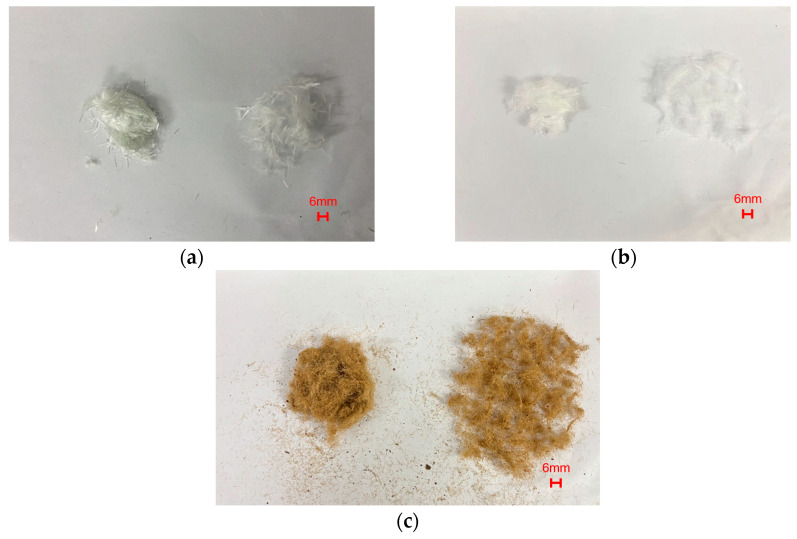
Fiber morphology: (**a**) glass fiber; (**b**) polypropylene fiber; (**c**) jute fiber.

This study selected three types of fibers: glass fiber (GF), polypropylene fiber (PPF), and jute fiber (JF) (their physical and mechanical parameters are shown in Table 2, and their morphology is shown in Figure 2). GF exhibits high tensile strength and elastic modulus, along with excellent heat resistance; however, it is prone to degradation in strongly alkaline environments. Therefore, alkali-resistant glass fiber or surface protection measures are typically employed to enhance durability when GF is used in high-alkali systems [40,41]. PPF is a polypropylene-based synthetic fiber characterized by low density and excellent chemical corrosion resistance, exhibiting stability in alkaline environments such as cement matrices. Its pronounced hydrophobicity makes it difficult to wet by cement paste, resulting in relatively poor interfacial bonding. Consequently, its reinforcement effect is significantly influenced by interfacial adhesion and fiber distribution [42,43,44]. JF is a natural woody cellulose fiber with strong hydrophilicity and pronounced moisture absorption. Moisture uptake may induce swelling of jute fibers, which can weaken the fiber-matrix interface and adversely affect the dimensional stability and mechanical performance under wet conditions [45]. Previous studies have shown that untreated jute fibers generally exhibit a relatively rough surface and may retain non-cellulosic constituents and surface impurities, whereas their removal by chemical treatment can improve fiber-matrix adhesion [46,47,48]. The SEM observations in this study are consistent with these reported characteristics. Such surface features may enhance mechanical interlocking, but they may also contribute to higher moisture sensitivity.

### 2.2. Methods

#### 2.2.1. Experimental Design

This study employed a two-stage experimental design to investigate the effects of gradation characteristics, represented primarily by *d*_50_ and accompanied by variations in *Cu* and *Cc*, as well as fiber type and dosage, on the performance of EICP-cemented RFA. Test indicators included CaCO_3_ content, specimen dry density, and unconfined compressive strength (UCS).

(1) Gradation Tests (Fiber-Free): Four gradation groups (ERFA1–ERFA4) with distinct *d*_50_ values were established, characterized as follows: ERFA1 (*Cu* = 7.30, *Cc* = 2.10, *d*_50_ = 1.14 mm), ERFA2 (*Cu* = 5.73, *Cc* = 0.80, *d*_50_ = 1.00 mm), ERFA3 (*Cu* = 4.27, *Cc* = 1.07, *d*_50_ = 0.83 mm), and ERFA4 (*Cu* = 3.35, *Cc* = 1.19, *d*_50_ = 0.67 mm). Each group included 3 parallel tests, totaling 12 groups.

(2) Fiber Modification Test: Based on the screening results from Phase I, the ERFA4 gradation was used as the reference (*Cu* = 3.35, *Cc* = 1.19, *d*_50_ = 0.67 mm). Three fibers (GF, PPF, and JF) were tested at dosages of 0.1%, 0.3%, 0.5%, 0.7%, and 0.9% (mass fraction, based on dry RFA mass). Three parallel tests were conducted for each combination, totaling 45 groups. In summary, this study completed a total of 57 test sets. For clarity, the sample series used throughout this study were denoted as ERFA (EICP-cemented RFA), BRFA (glass fiber-reinforced EICP-cemented RFA), JRFA (polypropylene fiber-reinforced EICP-cemented RFA), and HRFA (jute fiber-reinforced EICP-cemented RFA). These abbreviations are used consistently throughout the manuscript.

#### 2.2.2. Activity Test of Soybean Crude Urease

Changes in ion concentration in the solution during the enzymatic hydrolysis of urea lead to corresponding alterations in solution conductivity. Previous studies have demonstrated that under specific reaction conditions, changes in solution conductivity exhibit an approximately proportional relationship with the amount of urea hydrolyzed [49]. Therefore, this study indirectly characterized urease activity by measuring conductivity changes induced by urea hydrolysis. The soybean urease solution was mixed with urea at a volume ratio of 1:9, with a urea concentration of 1.35 mol/L. Under conditions of approximately 25 °C and pH 9.5, the conductivity change in the reaction system was measured using a portable conductivity meter (DDS-11A, Shenzhen Wanbang Environmental Technology Co., Ltd., Shenzhen, China) over a 10 min testing period. The initial value *N*_1_ and final value *N*_2_ were recorded. According to the calculation method proposed by Whiffin [50], urease activity was determined from the change in conductivity over a specified time period. The calculation formula is shown in Equation (1):

(1) $$U=\frac{\Delta N}{t}\times 11.11\times n$$

where *U* represents the urease solution activity (mmol·L^−1^·min^−1^); t denotes the conductivity sampling interval (min); Δ*N* indicates the change in conductivity (*N*_2_ − *N*_1_; mS/cm); and *n* signifies the dilution factor.

This experiment involved three parallel trials, yielding an average result of 12.24 mmol·L^−1^·min^−1^. Therefore, the selected urease concentration for this experiment was determined to be satisfactory.

#### 2.2.3. Sample Preparation

Under the condition of an initial void ratio (*e* = 0.75), the target loading mass was calculated based on the particle size distribution of each RFA group, and the test aggregates were prepared according to the mass percentage of each size fraction. Specimens were prepared as cylindrical columns of recycled fine aggregates (diameter 39.4 mm, height 78 mm) using split PVC molds. The upper end cap of the mold, connected to a grouting pipe, served as the grouting channel, while the lower base facilitated drainage. Before sample preparation, the RFA was washed, dried, and thoroughly mixed. Specimen loading was performed using a layered tamping method with four layers, each compacted sequentially to achieve the specified height and a level top surface. To prevent aggregate loss and reduce grout disturbance, thin geotextile sheets were placed at the top and bottom of the specimen (the bottom sheet prevented aggregate outflow, while the top sheet mitigates grout disturbance). Simultaneously, a cylindrical film was inserted between the specimen and the mold as a release layer for demolding. Fibers were incorporated at mass fractions of 0.1%, 0.3%, 0.5%, 0.7%, and 0.9% into the aggregate. The remainder of the preparation process was identical to that for unfibred specimens. To enhance fiber dispersion, an appropriate amount of distilled water was sprayed during mixing. After thorough mixing, the specimens were loaded.

**Figure 3:**
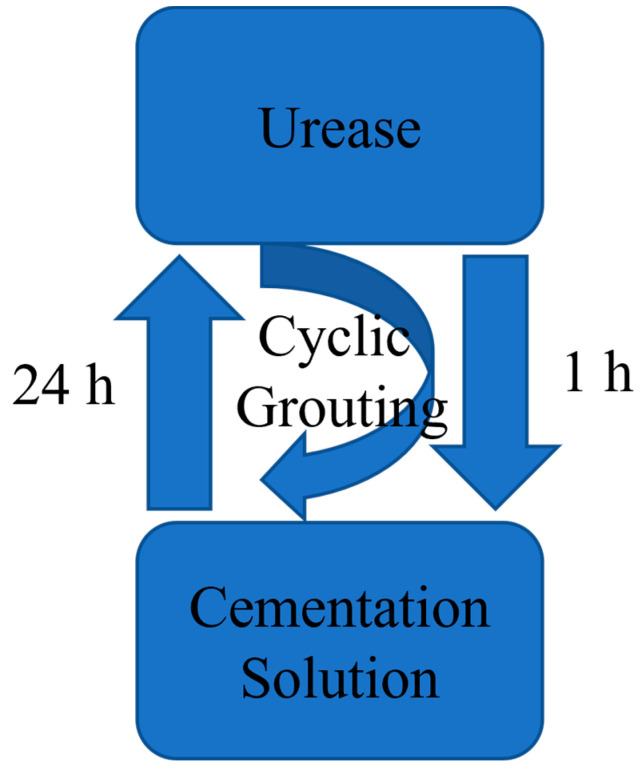
Grouting flow chart.

**Figure 4:**
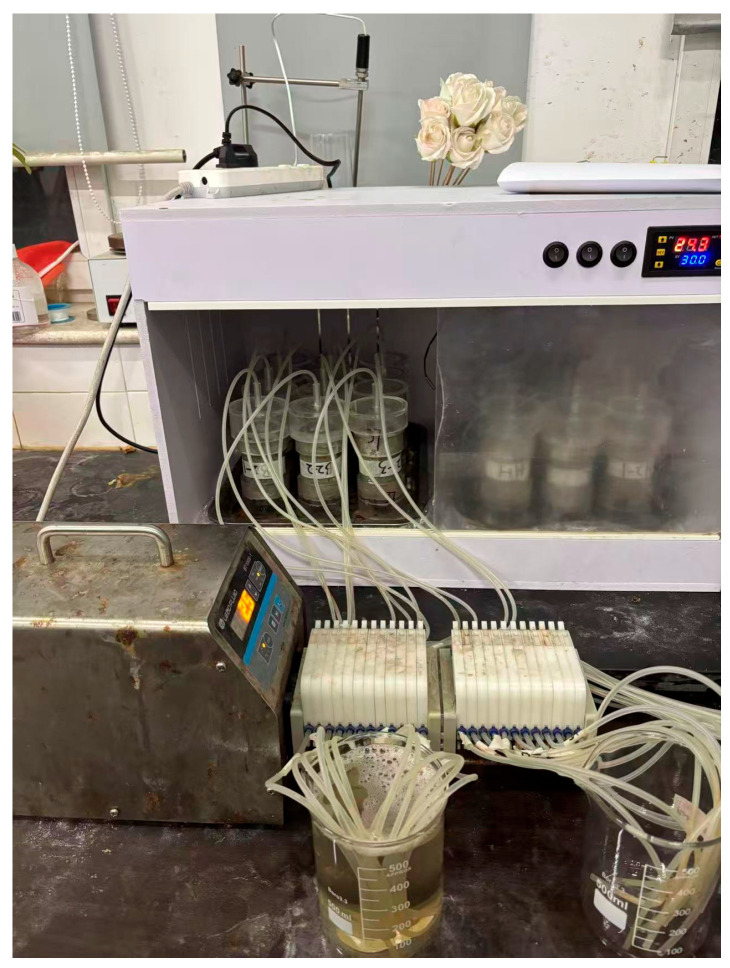
Grouting physical image.

**Figure 5:**
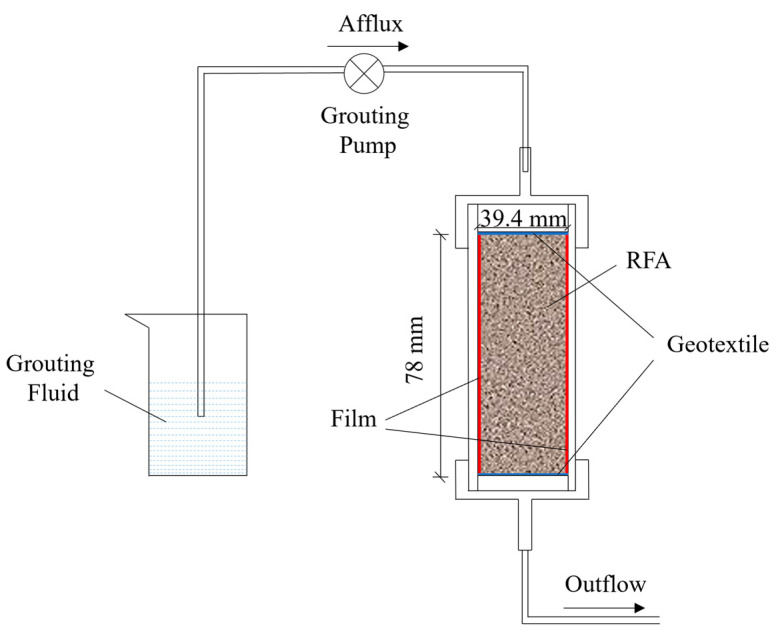
Grouting schematic diagram.

The EICP-induced calcium carbonate precipitation test employed a DHG-9075A grouting pump for stepwise cyclic grouting of specimens: First, distilled water was injected to continuously flush the specimen for 24 h, reducing the influence of soluble impurity ions; this was followed by the injection of 1 pore volume (1 PV) of urease solution and a 1 h static incubation to ensure thorough distribution of urease within the pores; subsequently, the pipeline was flushed to prevent premature reaction and blockage caused by residual urease reacting with the cementitious solution within the pipeline, followed by the injection of 1 PV of cementitious solution and a 24 h static incubation to complete the precipitation reaction (see Figure 3). Both the urease solution and grout were injected at a rate of 2.5 mL/min, with a total of 4 grouting cycles performed. To improve the axial uniformity of calcium carbonate distribution, each specimen was inverted 180° after each cycle before proceeding to the next treatment. Both the cyclic grouting and static reaction processes were conducted at a constant temperature of 30 °C. After all treatments were completed, the specimens were placed in a constant-temperature incubator at 30 °C for curing for 3 days. Photographs and schematic diagrams of the grouting process are shown in Figure 4 and Figure 5.

#### 2.2.4. Unconfined Compressive Strength Test

The unconfined compressive strength test was conducted using a CMT-5105GD universal testing machine (Zhuhai Sansi Taijie Electrical Equipment Co., Ltd., Zhuhai, China) in displacement control mode, set at a loading rate of 0.8 mm/min. Load–displacement data were recorded synchronously during testing and converted into the stress–strain curve. The unconfined compressive strength (UCS) of the specimens was determined as the peak stress on the stress–strain curve. After testing, raw data were exported for analysis, and photographs of failed specimens were taken for subsequent fracture morphology analysis. Detailed test results for specimens under different gradation schemes and fiber types and content ratios are presented in Table 3, Table 4, Table 5 and Table 6.

#### 2.2.5. CaCO_3_ Deposition Test

CaCO_3_ content was evaluated by a mass-gain method. The initial dry mass of recycled fine aggregate (RFA) was recorded as *M*_1_ (for fiber-reinforced specimens, *M*_1_ excludes the fiber mass). After EICP treatment, specimens were cured for 72 h, rinsed with distilled water, and oven-dried at 108 °C to constant mass (mass loss < 0.1%). For specimens without fibers, the constant dry mass was recorded as *M*_2_, and CaCO_3_ content was calculated as:

(2) $$\omega_{\left({CaCO}_{3}\right)}=\frac{M_{2}-M_{1}}{M_{1}}\times 100\%$$

For fiber-reinforced specimens, the constant dry mass was recorded as *M_3_*. The fiber mass (*M_f_*) was subtracted to obtain *M*_4_ (*M*_4_ = *M*_3_ − *M_f_*), and CaCO_3_ content was calculated as:

(3) $$\omega_{\left({CaCO}_{3}\right)}=\frac{M_{4}-M_{1}}{M_{1}}\times 100\%$$

where $\omega_{\left({CaCO}_{3}\right)}$ is the CaCO_3_ content (%), *M*_1_ is the initial dry mass of RFA (g), *M*_2_ is the constant dry mass after treatment for specimens without fibers (g), *M*_3_ is the constant dry mass for fiber-reinforced specimens (g), *M_f_* is the initial dry mass of fibers (g), and *M*_4_ is the corrected mass excluding fibers (g). Mass increase after MICP and EICP treatments has been widely used as a practical indicator of carbonate precipitation and deposition on recycled aggregates [51,52]. In the present study, the mass-gain method was adopted as an indirect approach to estimate newly formed mineralized deposits and was mainly used for comparative evaluation among different groups under identical treatment conditions. Therefore, the calculated values are reported as the apparent CaCO_3_ content and are hereafter referred to as “CaCO_3_ content” when no ambiguity arises. It should be noted that this method does not provide direct mineralogical confirmation of the precipitates, and the measured values may vary depending on the quantification method used [53]. Further confirmation by XRD, FTIR, or TGA is recommended in future work.

#### 2.2.6. Dry Density Test

Following the oven-drying procedure described in Section 2.2.5, the specimens were dried to a constant mass, denoted as *M*. For plain specimens, *M* = *M*_2_; for fiber-reinforced specimens, *M* = *M*_3_ (including fibers). The specimen diameter was measured at the top, middle, and bottom using a vernier caliper and averaged. The height was measured in three directions and averaged. The specimen volume *V* was calculated from the averaged dimensions. The dry density ρ was calculated as:

(4) $$\rho =\frac{M}{V}$$

where ρ is the dry density (g·cm^−3^); *M* is the constant dry mass of the specimen (g), i.e., *M*_2_ for specimens without fibers and *M*_3_ for fiber-reinforced specimens (including fibers); and *V* is the specimen volume (cm^3^).

#### 2.2.7. SEM Test

After drying, the EICP-cemented specimens were cut into blocks smaller than 1 cm × 1 cm × 1 cm. Before observation, the specimens were gold sputter-coated to enhance electrical conductivity. Scanning electron microscopy (SEM, JSM-7001F, JEOL Ltd., Tokyo, Japan) was used to examine the internal microstructural features of the treated samples.

## 3. Results and Discussion

### 3.1. CaCO_3_ Content and Dry Density

**Figure 6:**
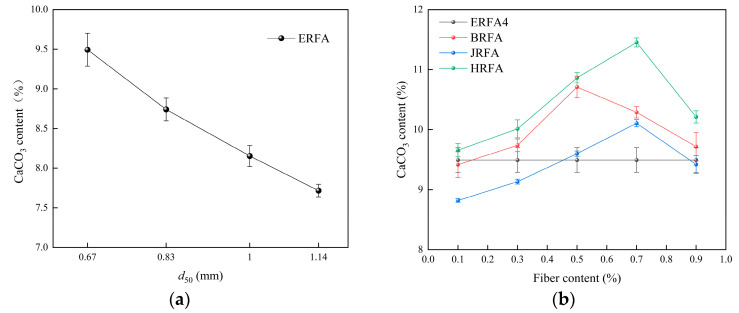
Variations in CaCO_3_ content of EICP-cemented recycled fine aggregate specimens: (**a**) effect of median particle size (*d*_50_); (**b**) effects of fiber type and fiber content.

As shown in Figure 6a, the CaCO_3_ content decreases monotonically across the tested gradation schemes, which are primarily differentiated by *d*_50_ and accompanied by variations in *Cu* and *Cc*. When *d*_50_ increased from 0.67 mm to 1.14 mm, the CaCO_3_ content decreased from 9.49% (ERFA4) to 7.72% (ERFA1), a reduction of 1.77 percentage points (a relative decrease of 18.65%). This indicates that within the gradation range studied, coarser ERFA is less conducive to CaCO_3_ deposition and retention. As *d*_50_ increases, the characteristic pore scale enlarges and flow pathways change. The most direct explanation is that the probability of stable deposition forming at particle contacts decreases, while the likelihood of detachment and migration under flow action increases. Microscopic evidence supporting the “differences in deposition distribution continuity and contact bridging” is presented in Section 3.5.

Figure 6b indicates that the CaCO_3_ content in all three fiber systems exhibits a unimodal variation with dosage, revealing a distinct optimal dosage. The peak CaCO_3_ contents were 10.71% at 0.5% for BRFA, 10.11% at 0.7% for JRFA, and 11.46% at 0.7% for HRFA. Compared to the unfilled fiber control group ERFA4 (9.49%), CaCO_3_ content increased by 12.86% (BRFA), 6.53% (JRFA), and 20.76% (HRFA) at their respective optimal fill levels. HRFA consistently exhibited higher CaCO_3_ levels, indicating that fiber surface characteristics and the number of interfacial anchoring sites play a crucial role in deposition efficiency. Notably, at 0.1% BRFA, CaCO_3_ content was slightly lower than ERFA4, potentially due to insufficient fiber dispersion at low loading levels, causing dispersed deposition distribution and failure to form effective deposits. Section 3.5 will validate this interpretation through SEM observations of fiber dispersion and interfacial deposition.

**Figure 7:**
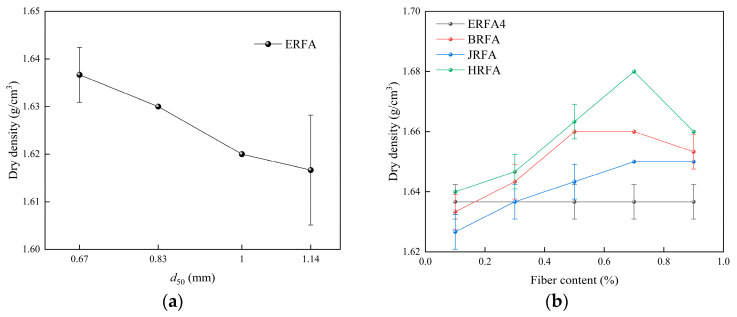
Variations in dry density of EICP-cemented recycled fine aggregate specimens: (**a**) effect of median particle size (*d*_50_); (**b**) effects of fiber type and fiber content.

Figure 7a shows that dry density decreases slightly across the tested gradation schemes as the representative *d*_50_ increases: ERFA4 (*d*_50_ = 0.67 mm) has a dry density of 1.637 g/cm^3^, while ERFA1 (*d*_50_ = 1.14 mm) has a dry density of 1.617 g/cm^3^, representing a decrease of 0.020 g/cm^3^ (a relative decrease of approximately 1.22%). The small variation in dry density indicates that, within the gradation range studied, changes in overall packing state are relatively limited, whereas variations in CaCO_3_ deposition levels are more pronounced. Combining Figure 6a with Figure 7a, specimens with higher CaCO_3_ content generally exhibit higher dry density, supporting the role of CaCO_3_ in enhancing bulk density through pore filling and surface coating. In summary, within the gradation range studied, dry density exhibits a weaker response to variations in gradation characteristics than to differences in CaCO_3_ deposition levels.

Figure 7b shows that fiber addition increases dry density with increasing dosage, reaching an optimum before plateauing or slightly declining at high dosages. Peak dry densities were 1.68 g/cm^3^ for HRFA at 0.7%, approximately 1.66 g/cm^3^ for BRFA between 0.5 and 0.7%, and approximately 1.65 g/cm^3^ for JRFA at ≥0.7%. Compared to ERFA4 (1.637 g/cm^3^), the dry density increases at peak addition levels were 2.63% (HRFA), 1.40% (BRFA), and 0.79% (JRFA). At low addition levels (0.1%), BRFA and JRFA exhibited slightly lower dry densities than the control group, potentially due to localized pore disturbance and insufficient dispersion introduced by the fibers. At high addition levels (0.9%), dry density gains tended toward saturation or decline, consistent with structural inhomogeneity and reduced deposition continuity caused by fiber bundling or agglomeration.

### 3.2. Unconfined Compressive Strength and Stress–Strain Behavior

**Figure 8:**
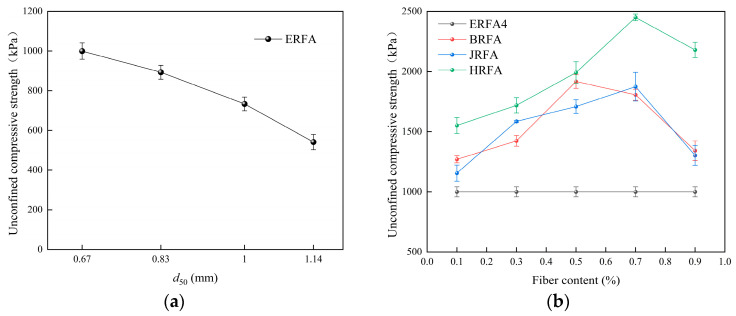
Variations in UCS of EICP-cemented recycled fine aggregate specimens: (**a**) effect of median particle size (*d*_50_); (**b**) effects of fiber type and fiber content.

Figure 8a shows that UCS decreases monotonically across the tested gradation schemes. ERFA4 (*d*_50_ = 0.67 mm) has a UCS of 1000 kPa, ERFA3 (*d*_50_ = 0.83 mm) has a UCS of 893 kPa, ERFA2 (*d*_50_ = 1.00 mm) has a UCS of 733 kPa, and ERFA1 (*d*_50_ = 1.14 mm) has a UCS of 541 kPa. The strength reduction from ERFA4 to ERFA1 is 459 kPa (a relative decrease of 45.9%). Considering that the dry density decreased by only 1.22% (see Section 3.1), this strength decline is more reasonably attributed to weakened spatial connectivity and force transmission continuity of the CaCO_3_ cementing matrix, rather than solely to differences in volumetric compaction. As further discussed in Section 3.5, SEM observations suggest that finer gradation schemes are associated with more continuous local deposition features and a greater number of particle-contact bridging structures.

Figure 8b indicates that the UCS of each fiber system exhibits a unimodal variation with dosage, with an optimal dosage present. The peak UCS values were 2450 kPa for HRFA at 0.70%, 1917 kPa for BRFA at 0.50%, and 1874 kPa for JRFA at 0.70%. Compared to ERFA4 (1000 kPa), the corresponding increases were 145% (HRFA), 91.7% (BRFA), and 87.4% (JRFA). Notably, the UCS increase far exceeded the increase in dry density (≤2.63%), indicating that the strength enhancement primarily resulted from (i) fiber crack bridging and energy dissipation and (ii) fibers promoting the formation of a more effective “fiber–CaCO_3_–particle” load-transfer network, rather than simple densification effects. When the fiber content increased to 0.9%, UCS decreased across all formulations. This is consistent with the interpretation that fiber bundling may reduce structural uniformity and promote more localized deposition features, thereby decreasing the number of effective load-bearing bridges.

**Figure 9:**
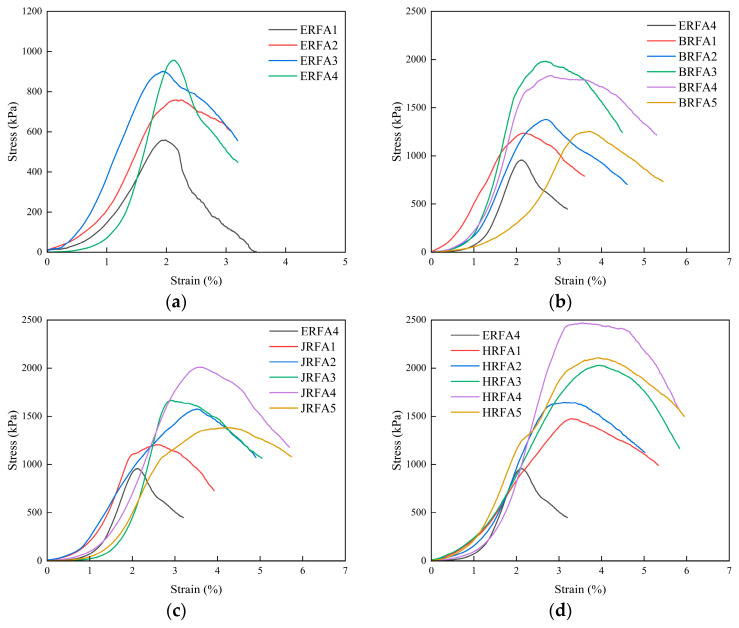
Unconfined compressive stress–strain curves of EICP-cemented recycled fine aggregate specimens: (**a**) effect of median particle size (*d*_50_); (**b**) effect of glass fiber content; (**c**) effect of polypropylene fiber content; (**d**) effect of jute fiber content.

Figure 9a shows that the curves for all groups undergo compaction, rapid stress increase, peak, and post-peak softening stages; no stable residual plateau appears post-peak, indicating rapid degradation of the load-carrying path after failure. The peak stress sequence across the different gradation groups aligns with the UCS pattern in Figure 8a. Post-peak decay rates varied between groups: ERFA2 and ERFA3 exhibited slower decay, while ERFA1 and ERFA4 showed steeper decay. This difference is closely related to variations in the spatial distribution of CaCO_3_ deposits and local connectivity.

Figure 9b–d show that fiber-reinforced specimens generally exhibit higher peak stress, greater peak strain, and milder post-peak softening, indicating that fibers simultaneously enhance both load-carrying capacity and ductility. An optimal fiber content exists, with performance improvements primarily stemming from bridging effects and interfacial mineralization networks rather than densification effects [54,55]. Compared with ERFA4 (peak stress ≈ 957 kPa; peak strain ≈ 2.11%), BRFA reached its optimum at 0.5% (≈1983 kPa; ≈2.69%), while JRFA and HRFA peaked at 0.7% (JRFA: ≈2010 kPa, ≈3.58%; HRFA: ≈2469 kPa, ≈3.57%), with HRFA showing the most pronounced post-peak load retention. It should be noted that Figure 8 presents the average UCS values from multiple tests, while Figure 9 displays representative stress–strain curves. Therefore, minor variations in peak values between the two figures are reasonable. The representative curves in Figure 9 were selected based on specimens for which the peak value fell within the reasonable fluctuation range of the group mean and exhibited no loading anomalies.

### 3.3. Relationship Between CaCO_3_ Content and UCS

**Figure 10:**
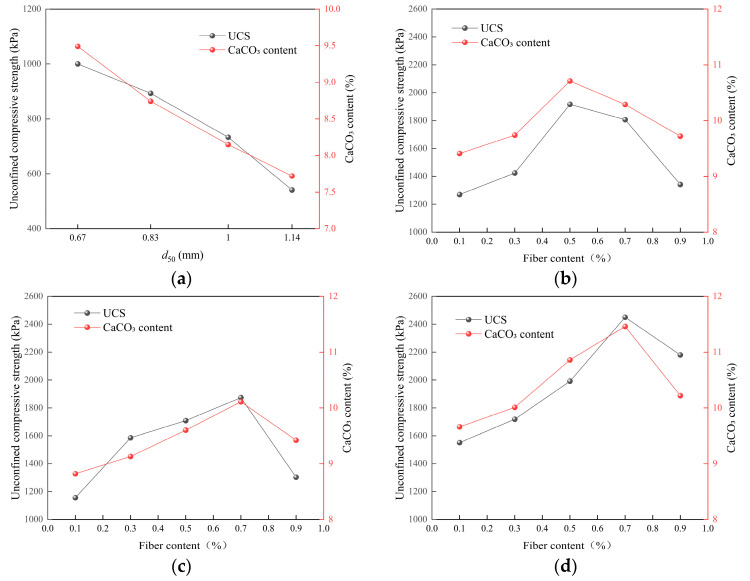
Relationships between UCS and CaCO_3_ content of EICP-cemented recycled fine aggregate specimens: (**a**) effect of median particle size (*d*_50_); (**b**) effect of glass fiber content; (**c**) effect of polypropylene fiber content; (**d**) effect of jute fiber content.

Figure 10a shows that both CaCO_3_ content and UCS decrease synchronously across the tested gradation schemes. This trend aligns with existing research: larger particle sizes alter pore scales and flow pathways, reducing effective CaCO_3_ deposition and cementing connectivity, thereby weakening strength [12,56]. Compared to ERFA1 (CaCO_3_ = 7.72%, UCS = 541 kPa), ERFA4 exhibited a CaCO_3_ content of 9.49% (a relative increase of 22.93%) and a UCS of 1000 kPa (a relative increase of 84.84%). The UCS increase significantly outpaced the CaCO_3_ content increase, suggesting that strength is more strongly influenced by the effective deposition pattern and connectivity of CaCO_3_ than by total deposition volume alone.

Figure 10b–d show that in all three fiber systems, both CaCO_3_ content and UCS exhibit unimodal variations with dosage: BRFA peaks at 0.5% (10.71%, 1917 kPa); JRFA and HRFA peak at 0.7% (10.11%, 1874 kPa; 11.46%, 2450 kPa), with both values declining when the fiber content increased to 0.9%. Thus, under the conditions of this study, the optimal contents for BRFA, JRFA, and HRFA were 0.5%, 0.7%, and 0.7%, respectively. Furthermore, even at similar CaCO_3_ content levels, UCS differences persist across fiber systems, further indicating that fiber cross-linking and interfacial mineralization nodes independently contribute to the restructuring of force transmission pathways (see Section 3.5). This also indicates that total CaCO_3_ content is not the sole determinant of strength; instead, strength is more sensitive to the effective deposition pattern and connectivity of the cemented structure. Previous studies have shown that UCS gains are more pronounced when deposition distribution is more uniform and connectivity is better [12,57,58].

### 3.4. Failure Modes of EICP-Cemented Specimens

**Figure 11:**
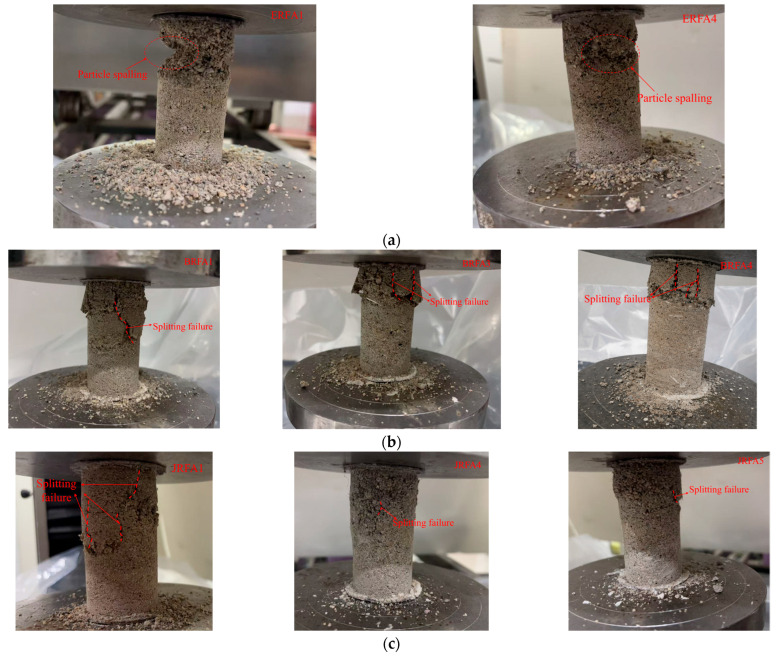

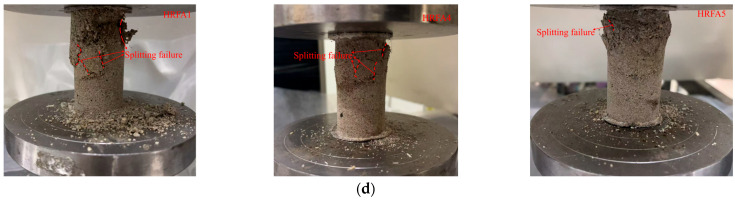
Failure patterns of EICP-cemented recycled fine aggregate specimens after unconfined compression: (**a**) different median particle sizes (*d*_50_); (**b**) glass fiber reinforcement with varying fiber contents; (**c**) polypropylene fiber reinforcement with varying fiber contents; (**d**) jute fiber reinforcement with varying fiber contents.

Figure 11a shows that failure in ERFA specimens under different gradation schemes primarily occurs near the loading end, manifesting as localized end crushing and particle spalling. No distinct through-shear cracks are observed, with end brittle spalling dominating the overall failure mechanism. Coarser specimens (e.g., ERFA1) exhibit more severe spalling and larger crushed zones, reflecting relatively insufficient binder continuity and effective bridging.

Figure 11b–d reveal that the failure mode of fiber-reinforced specimens evolves from end crushing and particle spalling to a composite failure pattern involving coexisting end crushing, particle spalling, and axial splitting cracks. With increasing fiber content, crack opening and penetration decrease, enhancing overall integrity after failure. Different fiber systems exhibit varying crack control effects: PPFs minimize crack opening and provide the most pronounced crack blunting; GFs reduce crack penetration but still exhibit significant end crushing; and JFs also inhibit crack propagation, though localized end crushing remains visible at high fiber content. Overall, fibers mitigate brittle failure through crack bridging and energy dissipation, while CaCO_3_ deposition and fiber-particle interfacial mineralization further enhance the continuity of the stress transfer network.

### 3.5. Microstructural Characteristics and Cementation Mechanism

**Figure 12:**
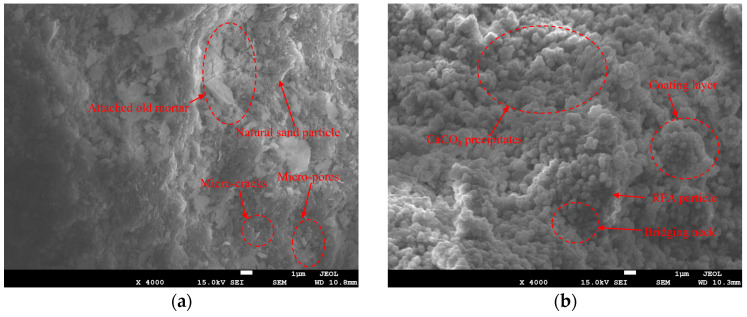
SEM images (×4000) of RFA before and after EICP treatment: (**a**) untreated RFA; (**b**) EICP-cemented RFA (ERFA).

As shown in Figure 12, the unbound RFA surface exhibits a distinctly heterogeneous composite structure: locally dense skeletal frameworks are surrounded by coarse, loosely adhered old mortar accompanied by pits and voids, indicating insufficient density and poor pore development in the old mortar. Adhesion of fine powder or powdered mortar further increases surface roughness and particle agglomeration. Micro-voids and micro-cracks are primarily concentrated in the old mortar and its transition zone, suggesting that the old mortar phase and the interfacial transition zone (ITZ) are potential weak points. Following EICP treatment, numerous fine granular deposits appeared on the ERFA surface, forming localized continuous coatings. This significantly mitigated the pitting and rough interface features, resulting in a more uniform and compact structure. Based on the CaCl_2_-urea-urease system and the observed mineralization morphology, these deposits can be attributed to EICP-induced CaCO_3_ formation. The cementation effect of CaCO_3_ is mainly reflected in two forms: surface coating, which covers and fills pore walls and voids, and intergranular bridging, which forms connecting necks at particle contacts and adjacent gaps, thereby enhancing interparticle bonding.

**Figure 13:**
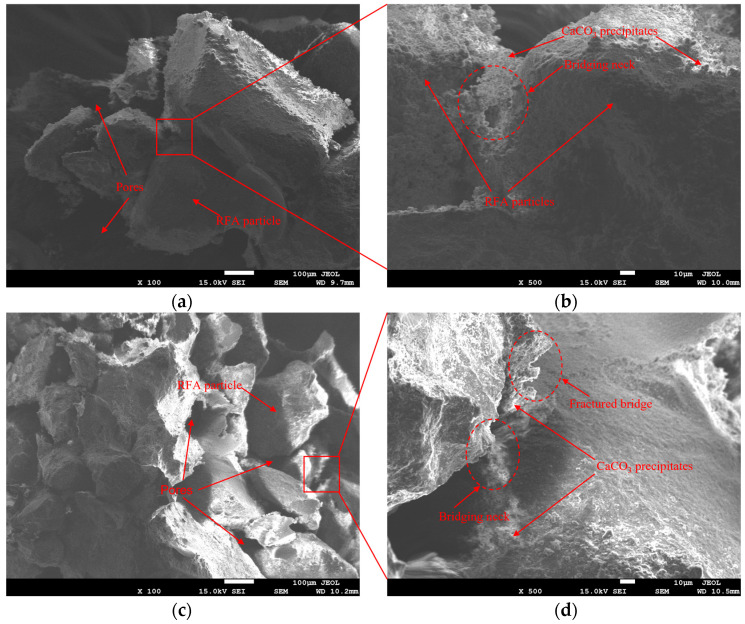
SEM images of EICP-cemented RFA with different *d*_50_: (**a**,**b**) ERFA1 at ×100 and ×500; (**c**,**d**) ERFA4 at ×100 and ×500.

Comparing specimens from different gradation schemes in Figure 13, CaCO_3_ coating and localized agglomeration can be observed in both cases. However, ERFA4 exhibits denser packing and smaller pores, and higher magnification reveals clearer bridging structures at particle contacts as well as localized fracture traces, which may indicate their possible mechanical involvement during loading. In contrast, ERFA1 shows more discontinuous surface coverage and localized agglomerate clustering, with fewer effective particle connections. These differences suggest that gradation affects the location of CaCO_3_ deposition and the effective mode of interparticle connection by regulating pore scale and flow pathways, thereby influencing the macroscopic cementation performance.

**Figure 14:**
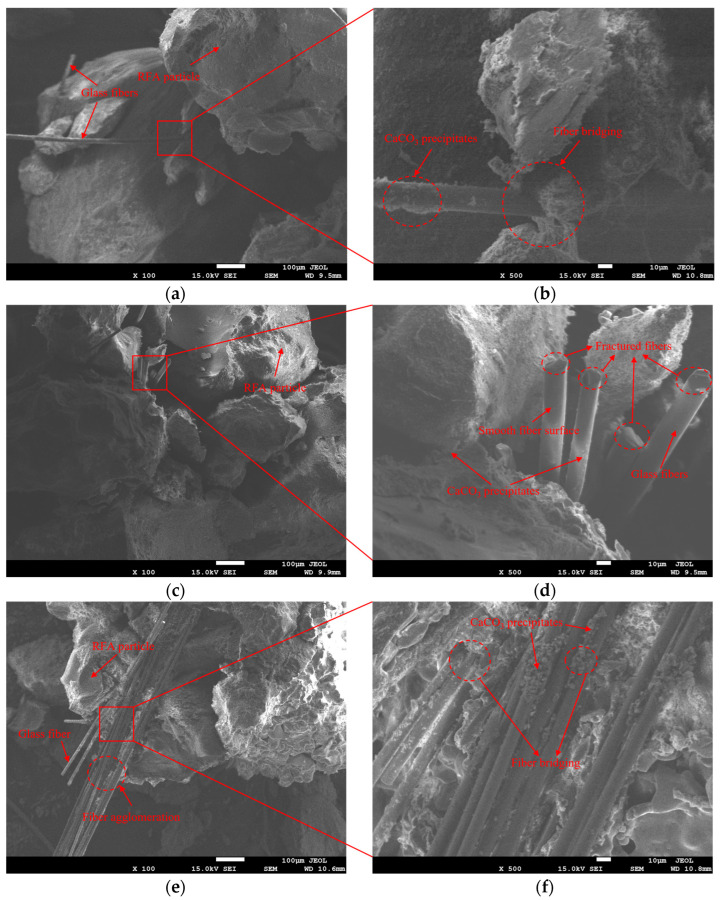
SEM images of glass-fiber-reinforced EICP-cemented RFA specimens at different glass fiber contents. (**a**,**b**) BRFA1 at ×100 and ×500; (**c**,**d**) BRFA3 at ×100 and ×500; (**e**,**f**) BRFA4 at ×100 and ×500.

**Figure 15:**
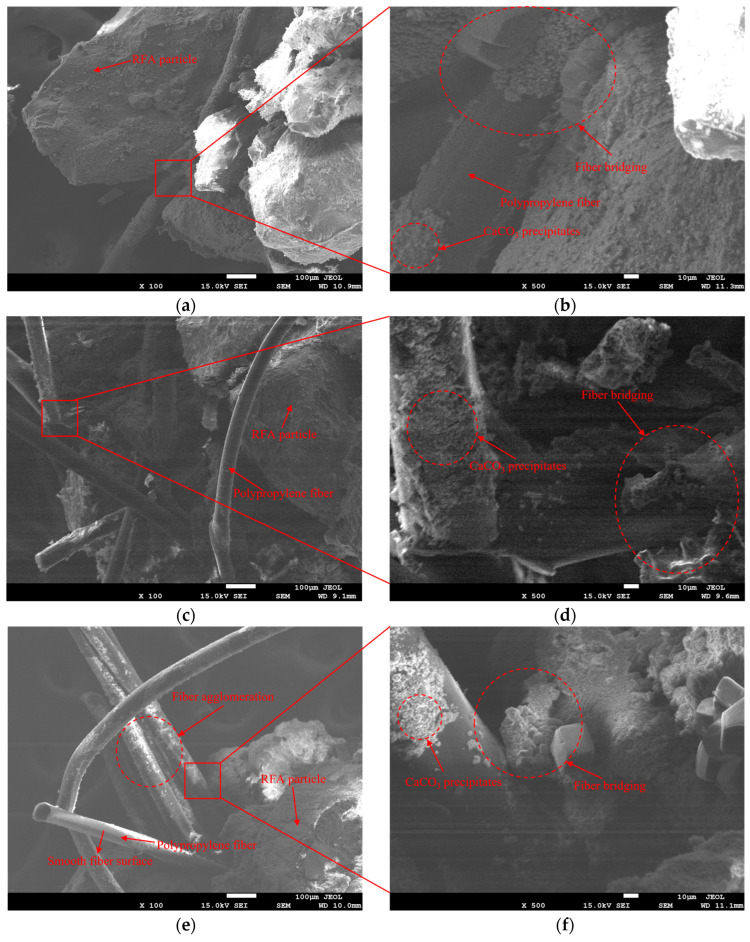
SEM images of polypropylene-fiber-reinforced EICP-cemented RFA specimens at different polypropylene fiber contents. (**a**,**b**) JRFA1 at ×100 and ×500; (**c**,**d**) JRFA4 at ×100 and ×500; (**e**,**f**) JRFA5 at ×100 and ×500.

**Figure 16:**
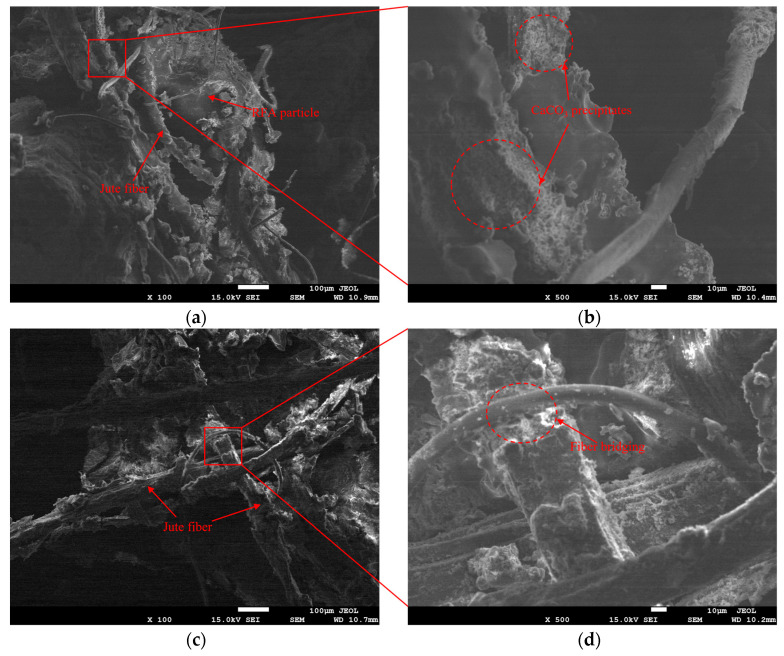

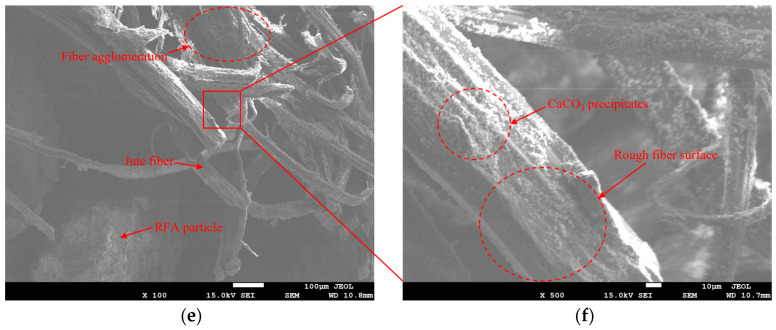
SEM images of jute-fiber-reinforced EICP-cemented RFA specimens at different jute fiber contents. (**a**,**b**) HRFA1 at ×100 and ×500; (**c**,**d**) HRFA4 at ×100 and ×500; (**e**,**f**) HRFA5 at ×100 and ×500.

In the fiber-synergistic EICP cementation system (Figure 14, Figure 15 and Figure 16), besides particle surface coating and interparticle bridging, CaCO_3_ can also deposit in the fiber–particle vicinity to form localized interfacial mineralization nodes. These features suggest the possible development of “fiber–CaCO_3_–particle” connection pathways, which may contribute to local stress transfer and energy dissipation.

For GF (Figure 14), low-magnification images (Figure 14a,c,e) show that fiber interweaving and overlap increase with fiber content. Samples with higher fiber content (BRFA3/BRFA4) exhibit more obvious aggregation, indicating dispersion limitations. High-magnification images (Figure 14b,d,f) further reveal deposits adhering to fiber surfaces and adjacent regions, locally forming bridging structures where fibers span pores. Given the relatively smooth surface of glass fibers (e.g., Figure 14d), their interfacial mechanical interlocking appears limited. Fiber anchoring and connectivity are more likely dependent on the interfacial bonding provided by CaCO_3_-mediated mineralization nodes. Signs of fiber fracture are observable in some areas (e.g., Figure 14d), suggesting that fibers may participate in stress transfer and energy dissipation during loading, rather than merely serving as inert fillers. At excessive dosage, fiber densification and localized agglomeration become more pronounced, while preferential deposition may reduce the spatial continuity of the cementing network. At the same time, dense fiber stacking may narrow local pore channels and potentially limit reactant migration within localized regions. This may lead to more localized CaCO_3_ deposition and lower overall cementation uniformity. This is consistent with the decline in macroscopic performance at excessive GF content.

For PPF (Figure 15), low-magnification images (Figure 15a,c,e) show that the low-content sample (JRFA1) contains relatively few fibers, mostly distributed in isolation. Near the optimum content (JRFA4), fibers interlace across pores and exhibit a more complete bridging geometry. Further increasing the content (JRFA5) results in more obvious overlap and localized agglomeration. High-magnification images (Figure 15b,d,f) show CaCO_3_ deposition on fiber surfaces and in nearby regions. Because the PPF surface is relatively smooth and provides limited anchoring sites, the deposits tend to form localized clusters and the potential connection points remain relatively dispersed. In JRFA4, mineralization nodes and bridging features at fiber–particle contact zones are more distinct, which may favor the formation of more continuous “fiber–CaCO_3_–particle” pathways. In contrast, JRFA5 shows more uneven deposition distribution, suggesting that excessive fiber content may reduce effective connectivity. In addition, bundling and agglomeration may restrict local pore channels and potentially limit local reactant movement and deposition spread. This may lead to lower deposition continuity and weaker macroscopic performance.

For JF (Figure 16), low-magnification images (Figure 16a,c,e) show fewer fibers with localized distribution in HRFA1; in HRFA4, interlocking increases and cross-pore bridging conditions become more favorable; HRFA5 exhibits more obvious bundling and localized agglomeration. High-magnification images (Figure 16b,d,f) reveal more pronounced CaCO_3_ deposition on fiber surfaces and in their immediate vicinity. The rough surface texture of jute fibers (e.g., Figure 16f) appears to provide additional nucleation and anchoring sites, enhancing interfacial mechanical interlocking among fibers, deposits, and particles. This feature may help explain the more evident interfacial mineralization characteristics and the relatively better macroscopic cementation performance observed at the optimal dosage (HRFA4). However, at higher dosage (HRFA5), bundling and agglomeration, together with more localized deposition, may reduce effective connection points and weaken connectivity. Concurrently, localized pore channel constriction caused by fiber bundling may introduce mass transfer limitations, causing CaCO_3_ deposition to favor localized enrichment rather than uniform distribution. This is consistent with the decline in macroscopic performance at excessive JF content.

## 4. Conclusions

This study investigates the influence of particle size distribution characteristics, represented primarily by median particle size *d*_50_ and accompanied by variations in *Cu* and *Cc*, as well as fiber synergistic effects, on the EICP cementation performance of RFA. Evaluations were conducted based on CaCO_3_ deposition, dry density, unconfined compressive strength (UCS), stress–strain response, failure mode, and microstructural morphology. Under the material and EICP process conditions employed in this study, the primary conclusions are as follows:

(1) Particle size distribution significantly influenced CaCO_3_ deposition. Across the tested gradation schemes, specimens with lower *d*_50_ values generally showed higher CaCO_3_ contents, indicating that coarser gradation schemes were less favorable for deposition retention and effective bonding.

(2) Dry density showed a weaker response to particle size distribution characteristics than CaCO_3_ deposition, although it followed the same overall trend. This suggests that CaCO_3_ deposition contributes to pore filling and surface coating, while its densification effect remains limited.

(3) UCS was more sensitive to variations in gradation characteristics than dry density, indicating that strength development was governed more by cementation network connectivity than by compaction alone. Across the tested gradation schemes, the reduction in strength was mainly associated with less favorable CaCO_3_ deposition locations, weaker effective crosslinking, and poorer spatial continuity of the bonding structure.

(4) Under the ERFA4 gradation scheme selected for fiber modification under the present test conditions, all three fiber types exhibited an optimal dosage window rather than a continuous strengthening effect with increasing content. Fiber incorporation generally improved both cementation efficiency and load-bearing capacity, whereas excessive fiber addition weakened the reinforcing effect. Among the tested fibers, jute fiber provided the most pronounced overall improvement.

(5) The beneficial effect of fiber reinforcement was mainly reflected in crack bridging, energy dissipation, and stress-transfer network formation, rather than in simple densification. Fiber-reinforced specimens showed higher strength, better deformability, and more gradual post-peak softening, together with a transition from relatively brittle local damage to a more distributed composite failure mode. Microscopic observations suggested that the synergistic interaction between fibers and CaCO_3_ deposition may promote the formation of a “fiber–CaCO_3_–particle” stress-transfer structure. However, when fiber content exceeded the optimal range, the reinforcing effect weakened because of fiber agglomeration, uneven deposition distribution, and reduced effective bonding connectivity.

It should be noted that the present conclusions are limited to the short-term performance observed under the current experimental conditions, since long-term durability was not directly evaluated in this study. Considering deposition efficiency, UCS enhancement, and post-peak ductility improvement, the optimal fiber content recommended under the present test conditions is 0.5% for GF, 0.7% for PPF, and 0.7% for JF. From an engineering viewpoint, the present results indicate that the EICP-fiber-treated RFA system may offer several potential advantages, including recycled fine aggregate utilization, reduced reliance on conventional cementitious binders, and improved strength and post-peak deformability. These features suggest possible relevance for low- to medium-strength applications, such as fill material stabilization, subbase improvement, or other recycled geomaterial systems.

However, since the present work is limited to laboratory-scale specimens and short-term testing, further study on durability, permeability, hydraulic response, and scale-up behavior is required before practical application can be considered. In particular, the feasibility of scale-up is expected to depend strongly on the control of reactant transport and the spatial uniformity of CaCO_3_ precipitation within larger-scale porous systems. At the field scale, heterogeneity in pore structure and flow pathways may lead to nonuniform deposition, localized clogging, or insufficient interparticle bonding, which could significantly affect the overall cementation performance. In addition, further characterization of permeability and flow behavior is recommended. Quantitative analysis of CaCO_3_ spatial distribution and bridging connectivity using micro-CT and MIP should be conducted to validate the proposed “mass transfer-deposition-connectivity” coupling mechanism, while the long-term durability of different fiber-reinforced EICP-cemented RFA systems should also be further investigated.

## Figures and Tables

**Table 1:** Gradation and physical properties of recycled fine aggregates (RFAs).

RFA	*Cu*	*Cc*	*d*_50_	*Gs*	*ρ_min_* (g/cm^3^)	*ρ_max_* (g/cm^3^)
RFA1	7.3	2.1	1.14	2.61	1.395	1.652
RFA2	5.73	0.8	1	2.60	1.445	1.666
RFA3	4.27	1.07	0.83	2.62	1.445	1.666
RFA4	3.35	1.19	0.67	2.62	1.447	1.651

Note: *C_u_* = *d*_60_/*d*_10_; *C_c_* = *d*_30_^2^/(*d*_10_*d*_60_); *d*_50_ denotes the particle size corresponding to 50% cumulative passing on the gradation curve; *G_s_* is specific gravity; *ρ*_min_ and *ρ*_max_ are the minimum and maximum dry densities.

**Table 2:** Physical and mechanical parameters of different fiber types.

Fiber Type	Color	Length	Tensile Strength	Density	Elastic Modulus	Melting Point	Elongation at Break
Glass fiber (GF)	White	6 mm	2000 MPa	2.699 g/cm^3^	85 GPa	750 °C	2.5%
Polypropylene fiber (PPF)	White	6 mm	400 MPa	0.91 g/cm^3^	3.5 GPa	160 °C	30%
Jute fiber (JF)	Light yellow	6 mm	320 MPa	1.3 g/cm^3^	/	/	3%

**Table 3:** Test Results of Physico-Mechanical Properties of EICP-Cemented RFA (ERFA).

Group	Specimen No.	*Cu*	*Cc*	*d*_50_	Initial Void Ratio (*e*)	CaCO_3_ Content (%)	Dry Density (g/cm^3^)	UCS (kPa)
ERFA1	ERFA1-1 ERFA1-2 ERFA1-3	7.3	2.1	1.14	0.75 0.75 0.75	7.67 7.67 7.81	1.63 1.61 1.61	497 558 569
ERFA2	ERFA2-1 ERFA2-2 ERFA2-3	5.73	0.8	1	0.74 0.74 0.74	8.04 8.30 8.12	1.62 1.62 1.62	747 759 694
ERFA3	ERFA3-1 ERFA3-2 ERFA3-3	4.27	1.07	0.83	0.75 0.75 0.75	8.59 8.75 8.88	1.63 1.63 1.63	855 900 924
ERFA4	ERFA4-1 ERFA4-2 ERFA4-3	3.35	1.19	0.67	0.75 0.75 0.75	9.73 9.34 9.41	1.64 1.63 1.64	1038 957 1004

**Table 4:** Test Results of Physico-Mechanical Properties of Glass Fiber-Reinforced EICP-Cemented RFA (BRFA).

Group	Specimen No.	Fiber Content (%)	*Cu*	*Cc*	*d*_50_	Initial Void Ratio (*e*)	CaCO_3_ Content (%)	Dry Density (g/cm^3^)	UCS (kPa)
BRFA1	BRFA1a BRFA1b BRFA1c	0.1 0.1 0.1	3.35	1.19	0.67	0.75 0.75 0.76	9.19 9.62 9.44	1.63 1.64 1.63	1237 1293 1281
BRFA2	BRFA2a BRFA2b BRFA2c	0.3 0.3 0.3	3.35	1.19	0.67	0.75 0.75 0.75	9.79 9.81 9.62	1.65 1.64 1.64	1425 1468 1378
BRFA3	BRFA3a BRFA3b BRFA3c	0.5 0.5 0.5	3.35	1.19	0.67	0.75 0.76 0.75	10.53 10.71 10.89	1.66 1.66 1.66	1880 1889 1983
BRFA4	BRFA4a BRFA4b BRFA4c	0.7 0.7 0.7	3.35	1.19	0.67	0.75 0.75 0.75	10.34 10.18 10.35	1.66 1.66 1.66	1834 1754 1833
BRFA5	BRFA5a BRFA5b BRFA5c	0.9 0.9 0.9	3.35	1.19	0.67	0.75 0.75 0.75	9.45 9.89 9.81	1.65 1.66 1.65	1254 1414 1359

Note: a, b, and c represent parallel test specimens under the same experimental condition.

**Table 5:** Test Results of Physico-Mechanical Properties of Polypropylene Fiber-Reinforced EICP-Cemented RFA (JRFA).

Group	Specimen No.	Fiber Content (%)	*Cu*	*Cc*	*d*_50_	Initial Void Ratio (*e*)	CaCO_3_ Content (%)	Dry Density (g/cm^3^)	UCS (kPa)
JRFA1	JRFA1a JRFA1b JRFA1c	0.1 0.1 0.1	3.35	1.19	0.67	0.76 0.75 0.75	8.79 8.82 8.85	1.62 1.63 1.63	1080 1181 1206
JRFA2	JRFA2a JRFA2b JRFA2c	0.3 0.3 0.3	3.35	1.19	0.67	0.75 0.75 0.75	9.18 9.12 9.10	1.64 1.63 1.64	1592 1588 1577
JRFA3	JRFA3a JRFA3b JRFA3c	0.5 0.5 0.5	3.35	1.19	0.67	0.75 0.75 0.75	9.58 9.57 9.65	1.64 1.64 1.65	1687 1666 1773
JRFA4	JRFA4a JRFA4b JRFA4c	0.7 0.7 0.7	3.35	1.19	0.67	0.75 0.76 0.75	10.1 10.17 10.05	1.65 1.65 1.65	1825 2010 1788
JRFA5	JRFA5a JRFA5b JRFA5c	0.9 0.9 0.9	3.35	1.19	0.67	0.75 0.75 0.75	9.35 9.59 9.32	1.65 1.65 1.65	1307 1383 1218

Note: a, b, and c represent parallel test specimens under the same experimental condition.

**Table 6:** Test Results of Physico-Mechanical Properties of Jute Fiber-Reinforced EICP-Cemented RFA (HRFA).

Group	Specimen No.	Fiber Content (%)	*Cu*	*Cc*	*d*_50_	Initial Void Ratio (*e*)	CaCO_3_ Content (%)	Dry Density (g/cm^3^)	UCS (kPa)
HRFA1	HRFA1a HRFA1b HRFA1c	0.1 0.1 0.1	3.35	1.19	0.67	0.76 0.75 0.75	9.55 9.65 9.77	1.64 1.64 1.64	1475 1583 1596
HRFA2	HRFA2a HRFA2b HRFA2c	0.3 0.3 0.3	3.35	1.19	0.67	0.75 0.74 0.75	10.10 9.84 10.10	1.65 1.64 1.65	1758 1645 1754
HRFA3	HRFA3a HRFA3b HRFA3c	0.5 0.5 0.5	3.35	1.19	0.67	0.75 0.75 0.75	10.82 10.81 10.96	1.66 1.67 1.66	2029 1889 2056
HRFA4	HRFA4a HRFA4b HRFA4c	0.7 0.7 0.7	3.35	1.19	0.67	0.76 0.75 0.75	11.53 11.45 11.38	1.68 1.68 1.68	2469 2463 2419
HRFA5	HRFA5a HRFA5b HRFA5c	0.9 0.9 0.9	3.35	1.19	0.67	0.75 0.75 0.75	10.24 10.10 10.30	1.66 1.66 1.66	2207 2107 2225

Note: a, b, and c represent parallel test specimens under the same experimental condition.

## Data Availability

The original contributions presented in this study are included in the article. Further inquiries can be directed to the corresponding author.
